# “They Don’t Respect Black Women”: Narrating a Young Black Woman’s Harrowing Birthing Experience

**DOI:** 10.1007/s40615-025-02467-w

**Published:** 2025-05-09

**Authors:** Jordyn Lally, Eniolufolake E. Ayoade, Robert E. Stadulis, Angela Neal-Barnett

**Affiliations:** Department of Psychological Sciences, Kent State University, 600 Hilltop Drive, Kent, OH 44242 USA

**Keywords:** Black pregnant women, Respect, Discrimination, Racism, Health care

## Abstract

Black women tend to feel underserved due to the lack of culturally competent care and support from healthcare systems. In particular, the combination effects of racial discrimination, inadequate prenatal care, and pregnancy complications contribute to the increased risks of stress and trauma among Black pregnant women. Though the literature has documented the challenges that Black women face in the healthcare system, in-depth narratives of birthing experiences may provide a better understanding of these issues. In this paper, we used a qualitative approach to describe the case study of a 30-year-old Black American woman who had a harrowing birthing experience. We conducted an individual interview with the participant upon her request. Six key themes were derived from her narrative that summarized her birthing experience: healthcare provider miscommunication, lack of control, trauma experience, interpersonal racism, systemic racism, and internalized racism. The participant had a birthing experience that she felt “could not go untold.” The discomfort and disrespect she experienced appear not to be uncommon in a considerable number of Black women of reproductive age. There is a need for healthcare providers to be trained to provide culturally appropriate care to Black women. In addition, healthcare providers need to treat Black women absent of prior stereotypes or biases to contribute to more effective care. Employing such treatment may improve Black women’s reproductive health-seeking behavior as well as maternal and child health.

## Introduction

Mothers’ experiences with medical professionals during their perinatal period can play a crucial role in shaping their physical, emotional, and psychological health. Positive interactions with medical professionals can foster trust and improve maternal outcomes, while negative experiences may contribute to heightened stress, anxiety, and long-term psychological consequences, including post-traumatic stress disorder (PTSD) [1–3]. Women who feel unheard or disrespected by their healthcare providers often struggle to voice their concerns, leading to decreased trust in medical care and poorer health outcomes [2, 4]. Negative interactions with healthcare providers may exacerbate the already stressful perinatal period for a woman, oftentimes leading to pregnancy complications and higher levels of distress, trauma, and poor health status [4, 5]. For Black women in the United States, these challenges are particularly pronounced, as Black women report disproportionately high rates of mistreatment and discrimination during pregnancy and childbirth, exacerbating racial disparities in maternal health [6].

Though there have been significant advancements in the awareness and reduction of racism-related issues in the US [7], there is still more to do to address racial inequities as they relate to maternal and child health. It is common for Black women to experience negative attitudes from healthcare workers, to be less informed about their health, to feel disrespected, and to have their pain undertreated [8, 9]. Such experiences leave them with little or no control over their health and limit their ability to make informed decisions about their health care. Further, research indicates that pregnant Black women frequently report that medical staff make light of their pain [8]. This was further expanded upon in a study which highlighted that Black women often report feeling ignored or dismissed by healthcare providers, which has led to delays in treatment and inadequate care [10]. These experiences characterize obstetric racism, which is described as the disturbing differential and stigmatizing treatment of individuals during pregnancy and postpartum by healthcare professionals based on race or ethnicity, including mistreatment, disregard for birthing wishes, neglect, dismissiveness, disrespect, intentionally causing pain, coercion, or degradation [10].

Black women are over two times more likely to experience maternal mortality than White women in the US [11]. Among the issues contributing to Black women’s likelihood of pregnancy-related deaths are lower healthcare quality, implicit bias, and racial discrimination during prenatal care and delivery [12–14]. Chronic stress due to racism and discrimination increases the likelihood of adverse maternal and infant health outcomes and higher mortality rates [8, 15]. Racism, in any of its forms, can significantly impact the childbirth experiences and pregnancy outcomes of Black American women. For instance, the experience of diverse forms of discrimination among pregnant Black women is linked with poor pregnancy outcomes, including low birth weight [5]. Moreover, enduring chronic stress due to racism can lead to physiological changes that may complicate pregnancy. Persistent exposure to racism may elevate and deplete stress hormones such as cortisol, which are associated with adverse birth outcomes [15]. Specifically, studies indicate that women who report experiencing racial discrimination are significantly prone to delivering low birth weight babies, having preterm births, and delivering babies who are small for their gestational age [16, 17]. As such, pregnant Black women are more likely to experience complications during pregnancy, such as preeclampsia, preterm birth, and low birth weight, all of which are associated with higher levels of stress and trauma [15, 18].

Further, the pervasive nature of racism, both systemic and individual, including those occurring within interactions in healthcare settings, further exacerbates the stressors on Black women, manifesting in various detrimental ways [16]. Furthermore, the lack of culturally competent care and support within healthcare systems often leaves Black women feeling marginalized and unsupported during pregnancy [19]. Culturally competent care involves healthcare practices that acknowledge and address the unique cultural, historical, and social contexts of patients, tailoring interventions to meet their specific needs. Examples include providing implicit bias training for healthcare providers, integrating community-based doula programs, and ensuring effective communication about medical procedures to build trust and respect [20, 21]. Altogether, the compounded effects of racial discrimination and inadequate prenatal care contribute to elevated risks of maternal and infant morbidities and mortalities [12, 16]. Thus, institutional or systemic racism has been associated with the receipt and quality of prenatal health care.

Trauma refers to a deeply distressing or disturbing experience that overwhelms an individual’s ability to cope, often leaving lasting emotional and physiological effects [22]. Stress, while a natural response to external pressures, can have negative health consequences when prolonged, particularly in environments marked by racism and discrimination. Distress is the subjective emotional discomfort resulting from exposure to stressors or trauma and can impair decision-making, emotional regulation, and overall well-being. PTSD emerges as a clinical condition when individuals experience prolonged and severe reactions to trauma, such as intrusive thoughts, hypervigilance, and emotional numbness [23]. For Black pregnant women, these concepts are intricately connected to navigating systemic racism, inadequate health care, and medical neglect, often compounding the emotional toll of pregnancy. These definitions underscore the importance of understanding and addressing the specific emotional and psychological challenges faced by Black women to inform trauma-sensitive and culturally relevant treatment during the perinatal period.

Critical race theory (CRT) provides a valuable lens for understanding racial disparities, emphasizing how structural and interpersonal racism shapes healthcare experiences and outcomes for Black women [24]. CRT demonstrates that racism is embedded within social structures and institutions, including health care, and that disparities in maternal health outcomes cannot be solely attributed to individual factors such as genetics or personal choices. Instead, systemic racism manifests through policies, implicit biases, and historical injustices that disproportionately disadvantage Black women. Studies have shown that Black women frequently report being dismissed by medical providers, having their pain underestimated, and experiencing poorer communication and care compared to their White counterparts [9, 19]. Consequently, CRT is particularly relevant in the context of obstetric racism.

Although quantitative studies have previously captured the problem of healthcare discrimination among Black perinatal women [5, 17], in-depth descriptions may be more beneficial in providing a deeper understanding of Black women’s perinatal experiences and making them feel more heard [19]. Quantitative methods, while useful in measuring disparities, often fail to capture the nuances of lived experiences, particularly when systemic issues like racism are concerned, limiting participants’ ability to express the full scope of their experiences [25]. Quantitative studies can be limited to standard questions, predefined categories, and fixed scores from numerical data. On the other hand, narratives can provide rich and detailed insight into individual perspectives by giving participants the opportunity to express their personal experiences in their own words and in a way that reveals the emotional, social, and contextual dimensions of issues that may be overlooked in numerical data [26]. Moreover, narrative approaches enable open-ended exploration that allows researchers to uncover emergent or novel themes that may not be easily captured within fixed-response quantitative approaches [25]. In addition, narratives humanize participants’ experiences, fostering an empathetic understanding of the issues while challenging stereotypes [27].

Systemic issues, such as racism, that are contextual and shaped by interactions, policies, and historical factors may be captured in narratives, offering an opportunity for detailed understanding of how systemic racism operates at both macro and micro levels [25]. Overall, narrative approaches may equip researchers and healthcare providers with substantial information to improve Black maternal and fetal health [10]. One way to attempt to provide a detailed narrative is to adopt the qualitative case study approach. A case study is an analysis of a real-world situation that may provide detailed narrative information, whether it involves an individual or a group [28]. Pregnant Black women do not often share detailed reports of their birthing experiences in research. Thus, information derived from such stories may provide insightful knowledge about treatment in healthcare settings and assist healthcare providers to better support Black women during their pregnancy and delivery while potentially improving Black maternal and child health. Consequently, the purpose of this study was to use the qualitative case study approach to examine the birthing experience of a Black perinatal woman, highlighting the systemic and interpersonal barriers she faced within the healthcare system. The aim was to use this narrative to shed light on the ways in which racial biases shape Black maternal health.

The findings are explained through the perspective of the CRT to provide deeper knowledge about the continued health inequities that Black perinatal women face. Although previous studies have used the qualitative approach to describe Black women’s negative perinatal healthcare experiences [29, 30], the current study uniquely highlights the worsened implications of distressing perinatal healthcare experiences on trauma, which is detrimental to the new postpartum mother and her newborn. By amplifying the participant’s voice, this study contributes to ongoing discussions on racial disparities in maternal healthcare and underscores the need for culturally competent, trauma-informed care that addresses both implicit bias and structural inequities. The participant in this case study was enrolled in a perinatal community research intervention. She felt sufficiently at ease to detail her experience with the intention that it be utilized to inform and inspire further research.

## Methods

### Participant Characteristics and Recruitment

The participant was a 30-year-old Black American married woman with a reported annual household income of less than $9999.00. At the time of data collection, she was using public health insurance. These attributes match those of mothers with negative birth outcomes in Cleveland, Ohio, which was the study locations [31]. No specific sampling procedure was utilized for this study. The participant was initially recruited for a community-based intervention facilitated by a non-profit organization that provides social and physical support for pregnant and postpartum mothers of color who face heightened risks of infant and maternal mortality. The intervention, known as Sister Circles, builds on long-standing traditions of collective healing, fictive kinship, and mutual community support among Black women. Originating from the Black Club movement [32], Sister Circles have been a cornerstone of Black female life for the past 150 years, fostering a sense of community and collectivism. Members often refer to one another as Sister X or Sister Y, emphasizing solidarity and interconnectedness [33, 34]. Inherently, Sister Circles provide Black women with help, support, knowledge, and encouragement [32, 34]. Over time, a Sister Circle can become a group of women experiencing the same health concerns who come together for education and support. The term Sister Circle has been used to designate support groups to reduce risk factors for chronic diseases [35]. Under this definition, the Sister Circle may be led by a professional (nurse, health educator, therapist) or by a survivor who has lived or is living with a particular health concern [20]. Thus, the intervention was designed to use culturally relevant cognitive behavioral techniques to teach expectant Black mothers to better learn how to recognize and positively manage stressors that play a role in infant mortality. Separate from the intervention, the participant requested that her birth experience be recorded in an interview by the first author who served as the project coordinator for the intervention. None of the researchers or coders had a prior relationship with the participant.

### Data Collection

The current study focuses on an individual who volunteered to tell her story to advocate and raise awareness of Black women’s treatment within the health system and call for improvements in Black maternal and child health. The interview was held in a private room at an intervention organizational site in Cleveland, Ohio, on June 20, 2018. At the participant’s request, an unstructured interview format was chosen, which allowed her to recount her childbirth experience freely and authentically in her own words. The participant notified the interviewer that she had an upsetting story that she wanted to share separate from the Sister Circle. The interviewer initiated the discussion by asking about the participant’s recent childbirth experience. The participant recalled her childbirth experience with little interruption from the interviewer. Follow-up questions were asked to clarify the details of her childbirth experience and elicit additional relevant information when necessary to ensure the accuracy and depth of the narrative. This included questions describing clinic staff roles in her experience or indicating the times that certain events occurred. The interview lasted 18 minutes and was audio-recorded with the participant’s consent. A trained undergraduate research assistant transcribed the interview recording verbatim for analysis. To ensure the trustworthiness of the data, the interviewer fostered a safe and supportive environment that allowed the participant to share sensitive details regarding her birthing experience. This opportunity allowed the interviewer to gain insight into the participant’s experience while ensuring that the participant’s experiences were captured without bias or misrepresentation during data collection and analysis. Because the participant appeared to have shared much personal and distressing information about her experience without any prompting, member checking was determined not to be necessary or ethical to minimize the risk of retraumatization or distress arousal.

### Ethical Considerations

This study was approved by the Kent State University Institutional Review Board. The participant gave written consent to be recorded and elected to remain anonymous. One main concern when collecting data from Black women in the US is their mistrust towards research participation, stemming from historical abuses [36]. This concern may have been alleviated given that the interviewer (a graduate psychology student) was a Bi-racial woman. This connection likely fostered trust, given the participant’s willingness to share her delivery story. The audio recording and transcript were stored in a password-secure computer, only available to the researchers, and used for research purposes alone.

### Data Coding and Analysis

Data were analyzed by inductive analysis, which involved coders reading through the data and identifying emerging themes without preconceived theories [37]. The coding procedure was performed manually in two stages. For the initial stage, three undergraduate research assistants (two Black females and one Black male) conducted open coding to identify emerging concepts in the data [37, 38]. They were prompted to independently read the transcript, highlight relevant quotes, and identify categories that represented the participant’s birthing experience. Coders were not provided with any specific themes or predetermined categories to identify in the transcript prior to open coding. Afterward, coders compiled and documented open codes, as they related to the participant’s birthing experience, in an Excel sheet.

The coders performed constant comparison when they met by comparing their open codes [39]. All open codes and corresponding quotes were compiled into a list across all coders. Open codes guided the next stage of focused or selective coding, where salient themes from the coded data were derived. In a consensus meeting, the coders compiled participant quotes and corresponding themes. After the coding process, it was apparent that data saturation was achieved when all coders and the coding supervisor (first author) reached a point at which there was unanimous agreement that no additional themes were evident in the transcript. Thereafter, themes agreed upon were documented as the final themes for this study. Coders discussed among themselves and selected final participant quotes for each theme. All three coders (100%) agreed upon which quote represented a specific theme. For one theme, two coders associated it with lack of control, whereas the third assigned a different name to the lack of control theme (see Table 1).

**Table 1:** Themes and definitions, identified statements, percentage of quotes, and coder agreement

Themes	Definitions	Number of quotes identified	Percentage of total quotes	Percentage of coder theme agreement
Health provider miscommunication	Poor communication and misunderstanding between patient and provider. Provider giving conflicting or inaccurate information to patients. Failure of provider to respond to a patient’s request for information	3	12.0%	100%
Lack of control	Any endorsement from the participant describing lack of control of their situation, plan, or desired outcome	4	16.0%	66.7%
Experience of trauma	Stressful events involving an actual or threatened death. Negative emotionality (crying) or thoughts (thoughts of dying, fear, helplessness) in response to trauma	11	44.0%	100%
Interpersonal racism	Prejudice and discrimination. Prejudice may manifest as differential assumptions about the abilities, motives, and intentions of others according to their race. Discrimination is described as differential actions toward others according to their race	3	12.0%	100%
Systemic racism	Institutionalized racism leading to differential access to goods, services, and opportunities of society by race	2	8.0%	100%
Internalized racism	Acceptance by members of the stigmatized race of negative messages about one’s own abilities and intrinsic worth. Characterized by not believing in others who look like them, and not believing in themselves. Can manifest as embracing of “Whiteness,” self-devaluation, resignation, helplessness, and hopelessness	2	8.0%	100%

## Results

The following sections present the findings, with representative narrative consensus quotes that illustrate each theme. Table 1 displays the six themes identified from the participant’s transcript, based on final selective coding. Each theme is defined, with the percentage of quotes represented by each theme and the percentage of theme agreement among coders. Below, a detailed description of the participant’s birthing experience is organized by linking corresponding quotes directly from the participant’s words to the derived themes.

### Hospital Visits and Diagnosis

According to the participant’s account, she visited the hospital for routine blood work, accompanied by her mother, on May 1, 2018, which was 11 days from her due date. Her mother, who noticed her swelling, suggested they visit the emergency room (ER) as a precaution. At the ER, she was informed that her blood pressure was slightly elevated but was discharged after being advised to check with the obstetrics section. Upon returning home, the swelling persisted, prompting another visit to the hospital the next day. Upon arrival at the hospital, she was diagnosed with preeclampsia, directed to the labor and delivery section, and advised to deliver her baby 10 days early. The consensus quotes, provided below, detail her accounts of emotions and experiences as she navigated interactions with healthcare providers during this period.

#### Healthcare Provider Miscommunication

The story begins with the participant recounting an interaction where she experienced miscommunication from her healthcare providers regarding her diagnosis of preeclampsia. According to her explanation, when she arrived the next day, before delivery, a midwife confronted her and questioned her decision to leave the hospital without further medical attention after her initial visit to the ER. The midwife was quoted as saying:

“I have a bone to pick with you… It says you left against doctor’s orders last night and didn’t go to OB, why not?... Haven’t you ever heard of the word pre-eclamptic before?”

The participant narrated that she did not fully understand the severity of her condition, as it had not been clearly communicated to her. She perceived the advice given to her as a precautionary measure rather than an urgent medical directive.

“Yes, but I was never aware that I had preeclampsia, because [the nurse] made it sound optional because she told me, ‘Your blood pressure’s a little high and your heart rate is a little elevated’, but she took it again and told me it was normal, and she wants me to check into OB as a precaution.”

Further, during critical moments of her labor and delivery, the participant described her experience of feeling additional distress due to what she perceived as a lack of clear communication. After undergoing an emergency cesarean section, she and her husband were reportedly left uncertain about the baby’s condition.

“[Husband is] sitting there crying because they still working on the baby. They put a ventilator over her, not telling us nothing was going on… And so, he thinks the baby over there dead. He’s sitting there with me; I told him to go over and check on her… They whisk her away, like I said, I was like ‘I don’t get to see her?,’ and that’s when I was like she’s dead, then she must be dead… I said she’s dead, that’s why they not—she’s dead, they couldn’t save her, that’s what my mind was saying, She’s dead, she’s dead, she’s dead.”

This perceived lack of communication during such a critical time contributed to the participant’s feelings of distress. She also reported feeling further discomfort due to the administration of medications, such as blood thinners after delivery, without what she considered adequate prior explanation.

“So, they was giving me all these blood thinners and I’m like, do you think, do I have a blood clot? Like, they didn’t even tell me, like, ‘We’re giving you this because of this, we’re giving you’—they were just handing me all this medicine and I’m just asking like, ‘What is this? What is this one for?’”

### Labor and Delivery

#### Lack of Control

The participant reported that the lack of information about her treatment added to her anxiety and contributed to a sense of losing control over her own medical care and birthing plan. She described instances where she perceived that she was no longer in control of the decisions regarding her health and her anticipated birth plan. These perceptions are illustrated in her narration of events. These findings are inferred from the quote below.

“You know, I was heavily sedated. I felt like I wasn’t in control of my body anymore. I wasn’t aware I was getting a C-section. I was told one thing, another thing happened. They took the baby away. I was never allowed to see her.”

The participant also reported an experience where an x-ray was necessary to ensure that instruments from the procedure had not been left inside her post-delivery, which added to her distress.

“I need to get the hell off this table. … she saying [and it was a Black woman] ‘I’m so sorry, I’m so sorry, we don’t usually do this, I’m not trying to hold you hostage in here’. I was like, well, can you tell the x-ray people…, so y’all can sew me up and I can go to bed?”

She reported not being allowed to see her newborn baby immediately after delivery, in addition to staff not granting her husband the privilege of cutting the umbilical cord.

“They never let me see her [the newborn baby]. They didn’t even put her over the curtain so I could see her. He [husband] didn’t get to cut the umbilical cord. He [husband] just happened to snap a picture so I can see what she looks like. And now I’m sitting there open, he’s sitting there crying because they still working on the baby. They put a ventilator over her, not telling us nothing was going on.”

#### Experience of Trauma

This theme was the most prevalent, representing 44% of the total quotes derived in this study. Specifically, the participant recounted a combination of events where she recalled feeling overwhelmed by intense fear for both her and her baby’s life, especially when she was rushed into the operating room for an emergency C-section due to complications.

“I said, I said, ‘Oh my God, I’m about to die, I’m about to die, I’m about to die, the baby’s about to die’. Like, I’m just trying not to cry, because I’m under all this anesthesia and now I’m really nervous.”

According to the participant, her husband also experienced distress during the emergency, particularly when he overheard alarming statements from the medical staff regarding the baby’s condition, which may have contributed to heightened fear and hopelessness.

“So now he’s [husband] crying because now he hears them say my heart rate is down, the baby’s heart rate is down, they can’t find the baby’s heart rate for four minutes, so he’s trying to hold my hand.”

She further recounted how the situation was exacerbated when she was left on the operating table for an extended period of time due to a recalled procedural oversight, which she found distressing.

“I’m still sitting there because they gave me an emergency C-section; they didn’t count all the instruments. So, the lady said ‘you have to give her an x-ray to make sure you didn’t leave anything inside of her’… every time I dozed off, they kept waking me up, because I’m under all this anesthesia, and they didn’t want me to die, I guess. So now I’m sitting here, open, on the table…”

This extended period of perceived vulnerability may have contributed to intensified symptoms of trauma. After the surgery, the participant described her distress over the separation from her baby and lack of information regarding the child’s condition.

“I just burst in tears like, I been up all morning, they haven’t let me see the baby, I don’t know if she alive’.”

According to her narrative, the trauma was further compounded by the perceived lack of immediate information about her baby’s condition. After the delivery, she reported that medical staff failed to provide timely updates, which led to further distress.

“The doctor comes in and apologizes. And I told her [the doctor], I said, ‘I’m very traumatized.’ Like, I was having nightmares. I said, ‘I didn’t sleep really well, I woke up thinking she was dead.’”

The baby survived. However, the participant stated that this traumatic experience may continue to influence her future decisions on childbearing. She reported that the stress and fears she experienced have contributed to her reluctance to consider having more children.

“It’s very traumatic because birth can go from zero to one hundred real quick… And it was traumatic, and you know, that made me not even think I would ever want to have children again because it just scared me too much.”

The participant indicated that the trauma from her birthing experience continues to affect her negatively.

“And now, even sometimes talking about it now, I still cry because … I just thought we were going to die…”

#### Interpersonal Racism

The participant expanded on her experience, sharing her perceptions of interpersonal racism encountered both before and after labor and delivery. During the interview, she opened her story with a powerful statement about the general lack of respect she felt as a Black woman navigating within the healthcare system.

“They don’t respect Black women.”

This statement prefaced several instances where the participant reflected on moments where she felt that healthcare professionals did not treat her with the dignity and respect that she deserved. For example, an event occurred during a night shift change when a nurse entered her hospital room, altering what she described as a previously supportive atmosphere. She indicated that this nurse addressed her husband in a manner that she perceived as impolite, failing to acknowledge him as her husband. She interpreted the nurse’s language as perpetuating negative assumptions and harmful stereotypes about young Black women.

“She referred to my husband as the baby daddy, ‘Hand it [the baby] to the baby daddy.’ He said ‘Excuse me? I’m her husband.’”

Furthermore, after being informed by her doula that she may have high blood pressure, the participant described her seeking medical advice. She described an encounter with a social worker whose questions, in her view, implied negative assumptions about her socioeconomic status and family structure.

“The social worker came and said, ‘Well we noticed you—you’ve been slacking on coming to your appointments, and you’re on our radar. So, we wonder, is there any kind of assistance to offer you to make sure that you get here? Do you have a car? Is the father involved?’’’

While it appeared that the social worker wanted to assist, the participant interpreted the manner in which these questions were asked as insinuating negligence on her part regarding her prenatal care. She recalled her response to the social worker, where she voiced her concerns.

“You trying to make it seem like I was a careless mother, and you know, I wasn’t. You should be talking to the midwife about communicating better with her patients.”

She further recounted the social worker’s response, which was perceived as reinforcing stereotypes against Black women.

“For your next child, you’ll know better.”

Reflecting on this interaction, the participant questioned the social worker’s assumptions regarding her plans to continue having children, which she felt was another example of a stereotype being applied to her. She replied:

“What makes you think I’m going to have more kids? Black women are not always out there making kids.”

#### Systemic Racism

A critical aspect of the participant’s story involved her perception that healthcare providers failed to provide adequate information about her and her baby’s conditions. The participant described this lack of communication as a significant issue, reflecting broader concerns she has about the healthcare system. She recounted instances where she felt insufficiently informed about the treatments administered to her, even after she inquired. For example, she noted that she was given various medications, including blood thinners, without being informed about their purpose or potential side effects.

“So, they was giving me all these blood thinners and I’m like, do you think, do I have a blood clot? Like, they didn’t even tell me, like, ‘We’re giving you this because of this, we’re giving you’—they were just handing me all this medicine and I’m just asking like, ‘What is this? What is this one for?’”

The participant expressed that this lack of communication left her feeling disempowered. She further described an incident where her mother expressed concern about the baby’s heart rate and felt that her concerns were dismissed by medical staff.

“My mother is offended, right, and my mother is scared now because she was like ‘We can’t find the heart rate, the doctors going to be mad.’”

The participant perceived this incident as indicative of a broader pattern where concerns raised by Black patients are downplayed or ignored, leading to feelings of neglect and inadequate care.

#### Internalized Racism

This story includes elements described as internalized racism stemming from the participant’s reflection on negative stereotypes toward Black women. She discussed her awareness of broader systemic issues that she believes Black women face in health care. She reflects on her experience, discussing how the lack of respect and support she encountered from healthcare providers may contribute to higher infant mortality rates occurring to children born to Black women.

“I told him, after all this, I see why Black women have the highest infant mortality rate. Because we don’t feel heard, we don’t feel respected, and we don’t feel helped.”

The participant also shared her thoughts on a statement made by a nurse, which she perceived to be a belief and stereotype against Black women’s attitudes toward their physical health.

“…And when she [the nurse] see Black women, they just assume we don’t care or they just assume these things about us.”

## Discussion

This study highlights the narrative of a young Black woman, who felt she faced significant disrespect, racial discrimination, and trauma during her childbirth experience. This participant’s story exemplifies the framework of critical race theory (CRT), which indicates racism being embedded in social institutions and shaping maternal healthcare experiences for Black women. Her experiences of feeling dismissed, misinformed, and disempowered are not isolated incidents, but rather reflect a broader system of healthcare inequities that affect Black women. CRT’s emphasis on structural racism is evident in how implicit biases, historical discrimination, and institutional policies converge and shape the healthcare inequities faced by Black women [24]. Additionally, the narrative reinforces intersectionality, a key CRT concept that recognizes how multiple identities (e.g., race, gender, socioeconomic status) interact to shape lived experiences.

The woman’s experience as a Black woman belonging to a lower income background highlights the compounding effects of racial and economic marginalization in healthcare settings. Concepts such as intersectionality underscore that Black women face unique vulnerabilities that cannot be understood solely through a singular lens of race or gender but must be examined through the interplay of multiple systems of oppression [40]. Moreover, this case study challenges mainstream healthcare discourses that often dismiss the realism of racial disparities. By documenting her experiences, this study amplifies voices that are frequently silenced in dominant medical narratives, reinforcing the need for systemic reform and culturally competent care. These findings indicate that addressing racial disparities in maternal healthcare requires systemic changes informed by CRT.

Despite national advancements in addressing racial disparities [7], we argue that our findings reveal that Black women in the US continue to perceive significant discrimination and marginalization within healthcare systems. This participant’s story is a stark reminder of the entrenched systemic and interpersonal racism that is perceived to persist in the US healthcare system, perpetuating health disparities. In this study, the participant’s opening statement summarized her birthing experience as one that portrayed disrespect for Black women.

The narrative highlights systemic issues through inadequate communication and the lack of transparency from healthcare providers. The administration of medications without proper explanations, including blood thinners, exemplifies disregard for patient autonomy and informed consent. The failure of the medical staff to disclose the magnitude of the participant’s state of preeclampsia portrays the minimization of serious health issues. The lack of communication was also reflected in the failure of staff to explain the status of the newborn’s health, heightening fear and uncertainty in the parents regarding the child’s condition. Such experiences reflect broader systemic issues where Black patients are not provided the same level of detailed medical information and respect as their White counterparts [41].

The present study illustrates the inability to be involved in decisions made regarding one’s healthcare needs. Performing a c-section without prior discussion, denying the new parents access to the baby without explanation, and not granting the father of the baby the privilege to cut the umbilical cord show disrespect towards the choice of a birthing experience. Although complications arose that necessitated the need for a c-section, proper communication is required to show respect for the patient’s birthing plan to prevent disappointments. Evidence reveals that unmet expectations with birthing plans are linked to PTSD, while following a birth plan may protect against PTSD [42]. These observations may be associated with the participant’s feelings of trauma during and after her birthing experience.

In the present study, the experience of trauma was the most prevalent among all themes. The participant’s childbirth experience was marked by significant trauma involving an unplanned emergency c-section, lack of communication about her baby’s health condition, and procedural oversights. These events contributed to her feelings of powerlessness and fear, which are critical components of psychological trauma. These findings are concerning, as trauma experienced during labor and delivery has been shown to disrupt mother-infant bonding and further exacerbate mental health issues. A study found that mothers who experienced traumatic childbirth often experienced disconnections stemming from psychological distress, including post-traumatic stress disorder, anxiety, and depression symptoms [43]. The study also found associations between traumatic childbirth and mothers struggling with negative feelings towards their infants, experiencing guilt and self-blame. The implications of these findings are important, as the stress and trauma experienced by mothers can interfere with their ability to respond sensitively to their infant’s needs, which may in turn adversely affect the child’s emotional development [43].

Research also indicates that traumatic experiences during childbirth are associated with long-term consequences, including chronic stress, anxiety, depression, and PTSD, where symptoms of flashbacks, nightmares, and hypervigilance were likely to be reported [23]. The prevalence of postpartum PTSD is notably higher among women who experience perceived threats to life, severe pain, or a loss of control during delivery, with approximately 9% of women developing PTSD after childbirth [23, 44, 45]. In the US, research shows that Black women experience higher rates of PTSD symptoms and reactions following childbirth when compared to White women [46]. This disparity can be compounded by the effects of systemic racism, inadequate healthcare support, and internalized negative stereotypes that heighten psychological distress and lead to poorer mental health outcomes in Black women [8, 15].

Further, interpersonal racism involves behaviors from dominant groups that harm people of marginalized racial groups, often through negative perceptions, assumptions, or attitudes [47]. The experience described in this study shows that interpersonal racism continues to exist in the healthcare system. The narrative reveals instances of disrespectful language and assumptions about the woman’s marital status, such as the healthcare professional referring to her husband as the “baby daddy.” Although it is unclear whether the staff intended it so, such language perpetuates stereotypes about young Black women having children outside of marriage [48]. Moreover, while the social worker appeared to address barriers to the participant’s attendance at her prenatal appointments, the participant sensed a tone from the worker of negligence and carelessness toward her health and her baby’s well-being. Such microaggressions not only undermine the dignity of Black patients but also diminish trust in healthcare providers, potentially deterring timely medical care, which can have severe implications on maternal and infant health outcomes [49, 50].

Systemic racism refers to institutional policies and practices that disproportionately disadvantage certain groups [12]. Systemic racism manifests within societal structures, including healthcare systems, and results in disparities in access, treatment, and outcomes, often leaving marginalized groups underserved. When inflicted on pregnant or postpartum women, this can be further referred to as obstetric racism within the health system [10, 30]. A previous qualitative study conducted among 31 pregnant women showed that Black pregnant women still experience racial discrimination, unfair treatment, and dismissed pain concerns in the healthcare system [29]. A more recent secondary analysis of a qualitative study also showed that Black women experience obstetric racism in terms of disrupted time with their newborns and lower quality of care [30]. Though the baby survived, the dismissal of concerns raised by the participant’s mother about the baby’s heart rate further illustrates systemic barriers that undermine the quality of care received by Black women, which increases the risks for adverse maternal and infant health outcomes. Findings from the present study corroborate previous studies demonstrating that Black women continue to experience high rates of mistreatment and discrimination during pregnancy and childbirth [6, 10, 29, 30].

Internalized racism occurs when individuals from marginalized racial groups accept and internalize negative stereotypes about their race, which may have lasting implications on their self-identity, self-esteem, and mental health [51–53]. The participant’s reflections demonstrate internalized expectations, as she reports how the perception of a healthcare provider’s lack of respect and support may contribute to higher infant mortality rates among the Black community. The narrative also suggests how societal views portraying Black women as unconcerned about their health result in implicit bias by healthcare workers, which is evidenced by their perceptions and interactions with Black pregnant women. Both internalized expectations and implicit bias may contribute to a larger narrative of pregnant Black women’s experiences in the US, in which their health concerns are often overlooked and dismissed [9, 10, 19]. In conclusion, prenatal and delivery care should be designed to ensure the safety of women and their children. The experience of the participant in this study has a likelihood of changing her decision regarding going to a hospital if she has a future pregnancy. This tendency is overall risky to maternal and child health.

### Implications

Findings from this study underscore the need for continued healthcare reform that is culturally sensitive and trauma-informed, especially for Black perinatal women. Culturally sensitive care emphasizes understanding and respecting the unique cultural backgrounds, beliefs, and needs of patients. Healthcare systems that integrate cultural competence training may reduce implicit biases and stereotypes, thereby enhancing the quality of care provided to marginalized groups. To address barriers to mental health services resulting from systemic racism and lack of culturally competent care, previous studies have equally highlighted the importance of this call to action, suggesting that culturally tailored prenatal care may mitigate disparities among Black perinatal women [21, 54]. Racially conscious providers have a greater tendency to better address the unique stressors Black women face, including the impacts of systemic racism and discrimination [21, 54]. The integration of community-based organizations into healthcare reform for Black postnatal mothers may also serve as trusted sources of support and advocacy, offering culturally relevant services that bridge the gap between healthcare providers and patients [55]. Community-based interventions empower mothers by educating them about their rights and available resources and advocating for systemic changes to improve maternal health outcomes. For example, the involvement of community health workers and doulas may help improve maternal and child health outcomes by providing physical, emotional, and informational support for perinatal women [46].

Furthermore, comprehensive postnatal care should include mental health interventions, or when necessary, trauma-informed care, to address the psychological needs of Black women who have experienced traumatic childbirth. This includes providing access to counseling, support groups, and other therapeutic resources to help mothers cope with trauma and strengthen their emotional well-being. Beck and Watson’s work emphasizes the importance of recognizing the signs and symptoms of trauma and integrating this knowledge into policies, procedures, and practices to avoid re-traumatization [43]. By adopting a trauma-informed approach, healthcare systems can better support Black mothers in their recovery and promote positive mother-infant bonding. Culturally tailored mental health services may improve psychological outcomes for Black women and foster healthy mother-infant relationships. Providing such services may help prevent the development of PTSD. Preventing PTSD from developing may be critical for the well-being of both mother and child. Future research may examine the possible causal pathway between obstetric racism and trauma.

In addition, training programs are needed to address implicit bias and the integration of community-based interventions into maternal healthcare. More broadly, public health policies and advocacies are required to address obstetric racism and improve Black maternal health care. As seen in this research, the negative effects of health disparities are not limited to the woman alone but can also affect her immediate family. Therefore, this narrative underscores the need for continued comprehensive reforms to eradicate racial inequities to ensure equitable treatment and health outcomes for all patients. It is also relevant to respect and listen to those accompanying pregnant women to delivery, as these are likely to be their most trusted sources of support and encouragement. Improved provider-patient communication and the involvement of perinatal women in their health care can enhance patient satisfaction. It is worthy of note that the study location, Cleveland, Ohio, is a major location where infant mortality remains a concern, with Black infants being two times more likely to die than White infants [56]. Therefore, programs to address social determinants of health among Black perinatal women are essential. The participant’s story began in the ER, suggesting that racism in health care exists at the crisis level. This finding has implications for improved policies regarding Black pregnant women visiting the ER. Finally, to address the systemic disparities in maternal health care and improve maternal and child health, health care for Black perinatal women should be equitable, compassionate, supportive, respectful, multifaceted, integrating culturally sensitive practices, and trauma-informed.

### Strengths and Limitations

This study is unique in some ways. First, the unstructured, open-ended nature of the interview allowed the participant the opportunity to narrate her story without limiting the depth of her responses or the possibility of response fatigue, which could have affected the quality of the results. Secondly, this study not only adds to previous research highlighting distressing perinatal experiences among Black women, but the study also emphasizes how these experiences can be traumatizing for the woman, which is ultimately unsafe for both the woman and the newborn. Whereas most studies focus on labor and delivery alone, this study includes the participant’s experience in the ER. Though this was a self-selected participant, her attributes match those of mothers in Cleveland, Ohio, who report negative birth outcomes [31].

Nonetheless, this study is not without limitations. We acknowledge that the findings are limited to one participant’s experience, representative of Black perinatal women in Cleveland, Ohio, and may not be generalizable to all pregnant Black women and all healthcare facilities in the US or elsewhere. Although the participant told her story less than 2 months after the occurrence, the narrative may have been subject to some level of recall bias. Moreover, it is unclear whether the participant’s perceptions match the intentions of the healthcare workers she encountered. Hence, the results should be interpreted with caution. Future studies may recruit more participants for similar interviews or conduct focus group discussions for more robust and generalizable findings. Still, the study provides an in-depth understanding of a key area that is relevant to decreasing the burden of maternal and child health. In addition, the participant did not indicate the racial identities of all healthcare providers she encountered other than suggesting that one of them was racist. Further clarification of this would have provided more understanding of the study. Lastly, there is a slight possibility that the experiences of pregnant Black women may have changed since the time we conducted this interview. Updated research is needed to examine the current situation on this topic using qualitative approaches to further uncover the lived realities of Black women and ensure that their voices influence healthcare policy and practice reforms.

## Data Availability

The redacted story is available upon request.
